# Suppression of steroid 5α-reductase type I promotes cellular apoptosis and autophagy via PI3K/Akt/mTOR pathway in multiple myeloma

**DOI:** 10.1038/s41419-021-03510-4

**Published:** 2021-02-24

**Authors:** Renjie Dou, Jinjun Qian, Wei Wu, Yanxin Zhang, Yuxia Yuan, Mengjie Guo, Rongfang Wei, Shu Yang, Artur Jurczyszyn, Siegfried Janz, Meral Beksac, Chunyan Gu, Ye Yang

**Affiliations:** 1 The Third Affiliated Hospital of Nanjing University of Chinese Medicine, Nanjing, China; 2 School of Medicine & Holistic Integrative Medicine, Nanjing University of Chinese Medicine, Nanjing, China; 3 Department of Hematology, Jagiellonian University Medical College, Cracow, Poland; 4 Division of Hematology and Oncology, Medical College of Wisconsin, Milwaukee, WI USA; 5 Department of Hematology, School of Medicine, Ankara University, Ankara, Turkey; 6 Key Laboratory of Acupuncture and Medicine Research of Ministry of Education, Nanjing University of Chinese Medicine, Nanjing, China

**Keywords:** Haematological cancer, Autophagy

## Abstract

Steroid 5α-reductase type I (SRD5A1) is a validated oncogene in many sex hormone-related cancers, but its role in multiple myeloma (MM) remains unknown. Based on gene expression profiling (GEP) of sequential MM samples during the disease course, we found that the aberrant expression of SRD5A1 was correlated with progression and poor prognosis in MM patients. In this study, the oncogenic roles of SRD5A1 were validated in human MM cell lines (ARP1 and H929) and the xenograft MM model as well as the 5TMM mouse model. MTT and flow cytometry were used to assess MM cell proliferation, cell cycle, and apoptosis post inducible knockdown SRD5A1 by lentivirus-mediated short-hairpin RNA (shRNA). Transcriptomic sequencing, immunofluorescence, and western blot were used to investigate the effects of SRD5A1 suppression on cell apoptosis and autophagy. Mechanistically, SRD5A1 downregulation simultaneously regulated both the Bcl-2 family protein-mediated apoptosis and the autophagic process via PI3K/Akt/mTOR signaling pathway in MM cells. Meanwhile, the autophagy inhibitor (3-methyladenine) and SRD5A1 inhibitor (Dutasteride) were utilized to evaluate their anti-myeloma effect. Thus, our results demonstrated that SRD5A1 downregulation simultaneously regulated both the apoptosis and the autophagic process in MM cells. The dual autophagy–apoptosis regulatory SRD5A1 may serve as a biomarker and potential target for MM progression and prognosis.

## Introduction

Multiple myeloma (MM) is the second most common hematologic malignancy worldwide. According to statistics collected in China, the incidence of MM in 2016 was 1.60 per 100,000 person-years, with 1.84 per 100,000 person-years for males and 1.30 for females, respectively^1^. It is estimated that the number of new cases is over 30,000 in 2019 in the United States^2^. The therapies for MM have undergone great progress over the past years, including allogeneic hematopoietic stem cell transplantation (ASCT) and novel chemotherapies, such as proteasome inhibitors and immunomodulatory drugs^3^. However, MM is still incurable for the majority of patients who become refractory to treatment and ultimately relapse, thus further development of new therapeutic agents is warranted, such as specific inhibitors targeting activated oncogenes.

SRD5A1, a member of the steroid 5α-reductase family (SRD5A1, SRD5A2, and SRD5A3) converting testosterone to dihydrotestosterone (DHT), was recently reported to be aberrantly expressed in several sex hormone-related cancers, such as breast cancer, endometrial cancer, and prostate cancer^4–6^. SRD5A1 was mainly expressed in the skin, scalp, liver, and brain tissues, while SRD5A2 was predominantly found in androgen target organs such as the prostate and genital skin, but only SRD5A1 was upregulated in prostate cancers^7,8^. However, there is no report on the oncogenic role of SRD5A1 in MM progression, considering the unequal incidence rates between females and males of MM patients.

This study aimed to investigate the potential involvement of SRD5A1 during MM pathogenesis, and the evidence from in vivo and in vitro data demonstrated that SRD5A1-mediated MM cell autophagy via PI3K/Akt/mTOR signaling pathway and induced MM cell apoptosis through Bcl-2 proteins family in MM. Furthermore, we validated that Dutasteride, an SRD5A1 inhibitor, induced myeloma cells apoptosis in human myeloma cells and xenograft mouse models. Thus, SRD5A1 might be a considerable therapeutic target in the development of MM therapy.

## Methods

### Cell lines and cell culture

MM cell lines ARP1, OCI-MY5, and NCI-H929, which were kind gifts from Dr. Siegfried Janz (University of Iowa, Iowa City, IA, USA), were cultured in RPMI 1640 medium (Biological Industries, Beit Haemek, Israel) supplemented with 10% fetal bovine serum (FBS) (Biological Industries) and 1% penicillin/streptomycin (P/S) solution (Sigma-Aldrich, St. Louis, MO) under the condition of 37 °C in a humidified atmosphere of 5% CO_2_. HEK-293T and 5TMM3VT, which were donated by Dr. Wen Zhou (Xiangya School of Medicine, Central South University, Key Laboratory of Carcinogenesis and Cancer Invasion, Ministry of Education; Key Laboratory of Carcinogenesis, National Health and Family Planning Commission, Changsha, China), were cultured in DMEM medium (Biological Industries) containing 10% FBS and 1% P/S solution in 5% CO_2_ at 37 °C.

### Reagent

Caspase-3 (#9662, 1:1000), Cleaved caspase-3 (#9661, 1:1000), PARP (#9542, 1:1000), Bcl-xl (#2762, 1:1000), Bcl-2 (#2876, 1:1000), Bad (#9292, 1:1000), Bax (#2774, 1:1000), ATG-5 (#12994, 1:1000), ATG-7 (#8558, 1:1000), LC3A/B (#12741, 1:1000), Akt (#9272, 1:1000), p-Akt (Thr308) (#9275, 1:1000), p-mTOR (#2971, 1:1000), p-p70S6K (Thr389) (#9234, 1:1000) and β-actin (#4970, 1:1000) antibodies were obtained from Cell Signaling Technology (Danvers, MA, USA). SRD5A1 (#66329-1-Ig, 1:1000) and p70S6K (#14485-1-AP, 1:2000) were purchased from Proteintech (Manchester, UK). The p-PI3K (Tyr607) (#AF3241, 1:1000) and mTOR (#AF6308, 1:1000) antibody were purchased from Affinity (Cambridge, UK). SQSTM1/p62 (#P0067, 1:1000) antibody was obtained from Sigma-Aldrich (St. Louis, MO, USA) and goat Anti-rabbit IgG/Alexa Fluor 647 antibody (#bs-0295G-AF647, 1:500) was purchased from Bioss (Woburn, MA, USA). LY2940002 (#9901) were obtained from Cell Signaling Technology. Dutasteride (#A1659), hydroxychloroquine (HCQ) (#B4874) and 3-methyladenine (3-MA) (#A8353) were purchased from APExBIO (Houston, USA). Doxorubicin (#D1515) was purchased from Sigma-Aldrich.

### Western blotting

Briefly, whole proteins were extracted with RIPA Buffer (#89900, Thermo Fisher, Waltham, USA) with protease inhibitor cocktail (#20124ES03, Yeasen, Shanghai, China). The extracts were separated by SDS-PAGE and then transferred to 0.45-μm immobilon-P transfer membrane (#IPVH00010, Millipore, Darmstadt, Germany). Membranes were blocked with 5% skim milk for 1 h at room temperature followed by incubation with a primary antibody at 4 °C overnight. Then the blots were detected with HRP conjugated secondary antibody and visualized with the Super ECL Detection Reagent ECL (#36208ES60, Yeasen).

### Lentiviral gene transduction

The shRNA sequence against SRD5A1, that is CTCGAGTGCTGTTGACAGTGAGCGACCTGTACCTGTTATCAATATATAGTGAAGCCACAGATGTATATATTGATAACAGGTACAGGCTGCCTACTGCCTCGGAGAATTC, was cloned into TRIPZ vector (Thermo Fisher Scientific, USA), which provides inducible shRNA expression in the presence of doxycycline (Dox). Recombinant lentivirus was produced using transient 293T cell transfection. Transfected efficiency was verified by western blotting.

### Flow cytometry

In total, 1 × 10^6^ cells were fixed with 5 mL of cold ethanol at −20 °C overnight. After washed twice with cold PBS, the cells were suspended with 200 μl of PBS, and supplemented with 10 μl of RNase A stock (#10405ES03, Yeasen) solution and incubated 60 min at room temperature. After incubation, cells were stained with propidium iodide (PI, # 40710ES03, Yeasen) solution (40 μg/mL in PBS) for 15 min at room temperature and the cell cycle was analyzed using Flowsight flow cytometer (Merck Millipore, Darmstadt, Germany). For the cell apoptosis experiment, the ARP1-SRD5A1-KD and H929-SRD5A1-KD cells post 2 μg/mL treatment for 48 h at a density of 2 × 10^5^ cells were collected and stained using Annexin-V-Allophycocyanin (Annexin-V-APC) (#640941, BioLegend, San Diego, USA) and PI for 15 min in dark conditions, and the apoptosis of the cells was detected using flow cytometer.

### Cell viability assay

Cells were enumerated by Trypan blue staining using a hemocytometer (Qiujing, Shanghai, China), then were seeded into 96-well plates at the density of 3000–4000 cells/well. After treatments, cells were incubated with MTT for 4 h, then the supernatant was removed, and the formazan crystals were dissolved in 200 μL dimethyl sulfoxide (DMSO), the optical density was measured at 570 nm.

### Transcriptomic sequencing

Cells were pelleted, and RNA samples were isolated and sent to the MicroAnaly (Shanghai, China) for constructing RNA-seq library. Briefly, total RNAs were isolated, and mRNA was enriched and then were pooled together for cDNA synthesis and sequencing. The transcriptomic RNA sequencing was performed on an Illumina NovaSeq 6000 platform, to create paired-end reads with a length of 150 bp (PE150). The differentially expressed genes (DEGs) were identified using edgeR (|logFC | >1 & FDR < 0.05), and these DEGs were undergone Gene ontology and KEGG pathway analysis. The original raw data and detailed processed data were submitted into Gene Expression Omnibus (GSE155858).

### Transmission electron microscopy

Cells were harvested by scraping them from the plates. They were then washed twice with PBS and fixed with 2% paraformaldehyde/2% glutaraldehyde in 0.2 M sodium cacodylate buffer (pH 7.4). Cell pellets were postfixed with 1% (v/v) osmic acid in sodium cacodylate buffer and were then stained with 1% uranyl acetate. Following dehydration, pellets were embedded in Durcopan (Durcopan, Fluka Chemie, Buchs, Switzerland). Ultrathin sections were prepared using an ULTRACUT S ultramicrotome and were observed with a JEM 1010 transmission electron microscope.

### Immunofluorescence

Cells were collected by cytospin on glass slides and fixed with 4% paraformaldehyde for 15 min. After washing in 10% PBS, the slides were permeabilized with 0.2% Triton X-100 in PBS and then were blocked with 4% bovine serum albumin (#A8020; Solarbio, Shanghai, China) for 1 h at room temperature. After incubated with LC3A/B antibody (#12741, 1:1000, Cell Signaling Technology, Danvers, USA) overnight at 4 °C, the slides were washed with PBS and then incubated with Alexa Fluor 647s antibody (#bs-0295G-AF647, Bioss, Woburn, USA). The cells were stained with nuclear dye DAPI (#C0080; Solarbio, Shanghai, China). All the images were captured with the fluorescence microscope, and representative images were shown.

### Myeloma xenograft tumor mouse model and 5TMM mouse model

MM cells (1 × 10^6^) of OCI-MY5 and OCI-MY5-SRD5A1-OE were injected subcutaneously into the right and left flank of 6–8 weeks’ male/female NOD. Cg-Rag1 (NSG) mice (Vital River Laboratory, Beijing, China) (*n* = 8), respectively. The tumor volume was measured with a caliper every 3 days to evaluate the tumor growth rate. Once tumors reached 15 mm in diameter, mice were sacrificed by CO_2_ asphyxiation.

The 5TMM3VT murine myeloma cells (1 × 10^6^) were injected intravenously into the abdomen of 6-week-old male/female C57BL/KaLwrij mice (Jackson Laboratory, Bar Harbor, ME) (*n* = 14). After 3 days, SRD5A1 inhibitor Dutasteride treatment used on seven mice and intraperitoneally injected at the dose of 25 mg/kg three times a week until all the mice were dead. The time of death caused by paralysis in the control group and the Dutasteride group was recorded in turn. All animal work was performed in accordance with the Guide for the Care and Use of Laboratory Animals (National Institutes of Health, USA) and approved by the Animal Ethical and Experimental Committee of Nanjing University of Chinese Medicine (ACU-15 & ACU170501).

For additional detailed methods, please refer to the Supplementary Materials and Methods.

### Statistical analysis

All data were shown as means ± SEM and performed using the GraphPad Prism program. Multiple groups were analyzed by one-way ANOVA, and paired groups were analyzed by Student’s *t* test, the survival data were plotted using Kaplan–Meier curve and analyzed by log-rank (Mantel–Cox) test. *P* < 0.05 was considered as significant.

## Results

### Increased SRD5A1 expression is correlated with poor survival in MM patients

**Fig. 1:**
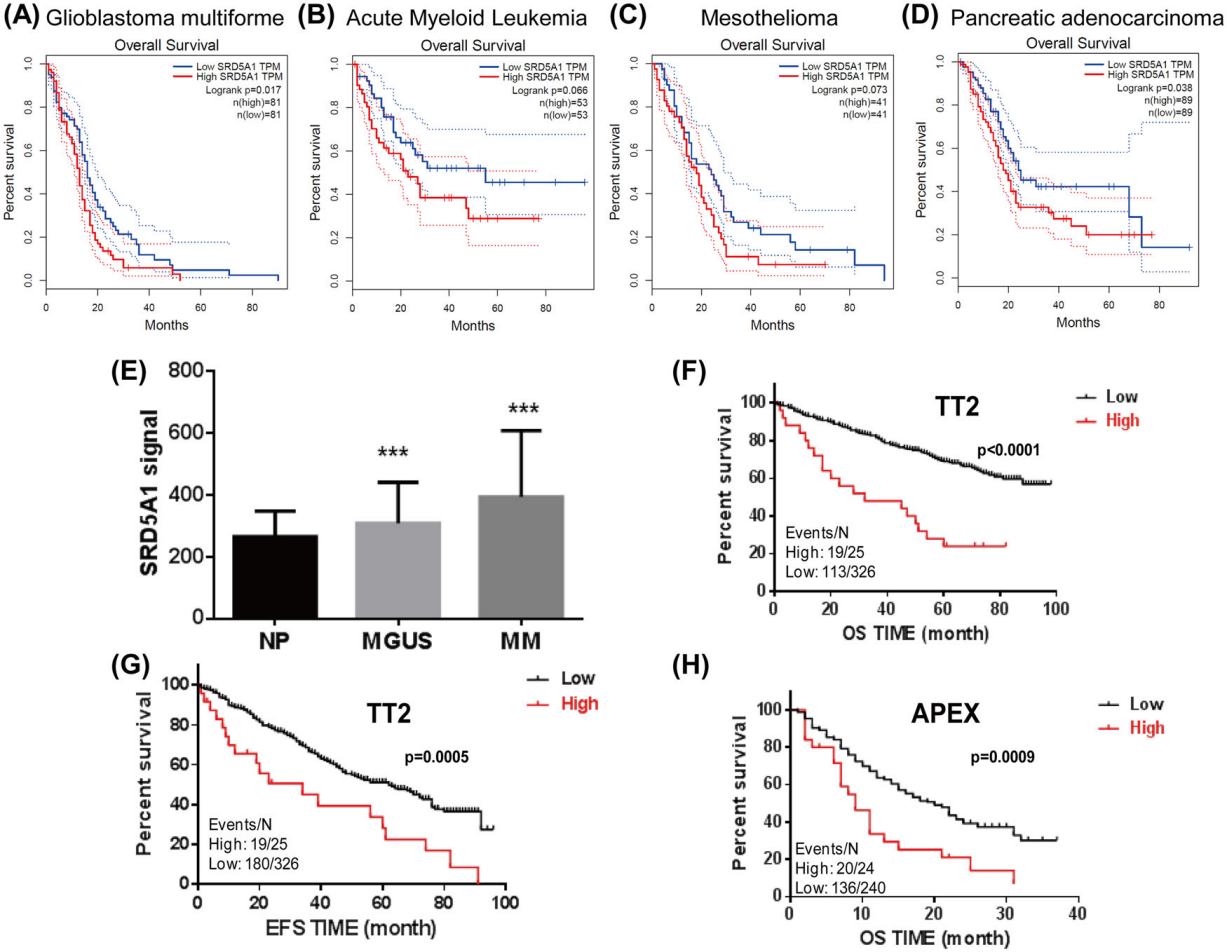
SRD5A1 is a poor prognostic marker in MM. **A**–**D** Kaplan–Meier analysis of OS time, revealing that high SRD5A1 expression conferred inferior outcomes. **E** Histogram depicted the SRD5A1 signal in NP, MGUS, and newly diagnosed MM patients. **F**–**H** Kaplan–Meier analysis was performed based on SRD5A1 expression in different cohorts, the MM patients with high SRD5A1 expression had a significantly inferior OS and EFS time, and OS time was also analyzed in the APEX clinical trials. *P < 0.05, **P < 0.01, ***P < 0.001.

To understand the clinical outcome of SRD5A1 expression, we examined the data about multiple cancers from the Cancer Genome Atlas (TCGA) using GEPIA online tool with customizable functional analysis, such as tumor/normal differential gene profiling and patient survival analysis^9^. The Kaplan–Meier analysis demonstrated that high SRD5A1 level was associated with poor prognosis for multiple cancer patients, such as glioblastoma multiforme, acute myeloid leukemia, mesothelioma, pancreatic adenocarcinoma, etc. (Fig. 1A–D and Supplementary Fig. S1A–C), and SRD5A1 was almost exclusively upregulated in tumor tissues relative to normal counterparts (Supplementary Fig. S1D, E). Moreover, we further explored the detailed expression of SRD5A1 in plasma cells of MM patients during different disease stages using datasets from NIH Gene Expression Omnibus (GSE2658). As shown in Fig. 1E, the expression of SRD5A1 was significantly increased successively from normal people (NP), monoclonal gammopathy of undetermined significance (MGUS) to MM patients. In addition, MM patients bearing high SRD5A1 expression suffered poor prognosis compared to low SRD5A1 expression patients in TT2 (Total Therapy 2) cohort.

Furthermore, high SRD5A1 expression was linked to shorter response duration of both event-free survival (EFS) and overall survival (OS) (Fig. 1F, G). As shown in Fig. 1H, we further tested SRD5A1 mRNA expression in MM patients from phase III APEX (assessment of proteasome inhibition for extending remissions) trials which evaluated the response to standard therapies (bortezomib or dexamethasone), supporting that SRD5A1 might be relevant to myeloma relapse^10^. Taken together, we proposed that SRD5A1 might function as an oncogene in MM.

### Knockdown of SRD5A1 induces growth suppression, cell cycle arrest, and apoptosis in MM cells

**Fig. 2:**
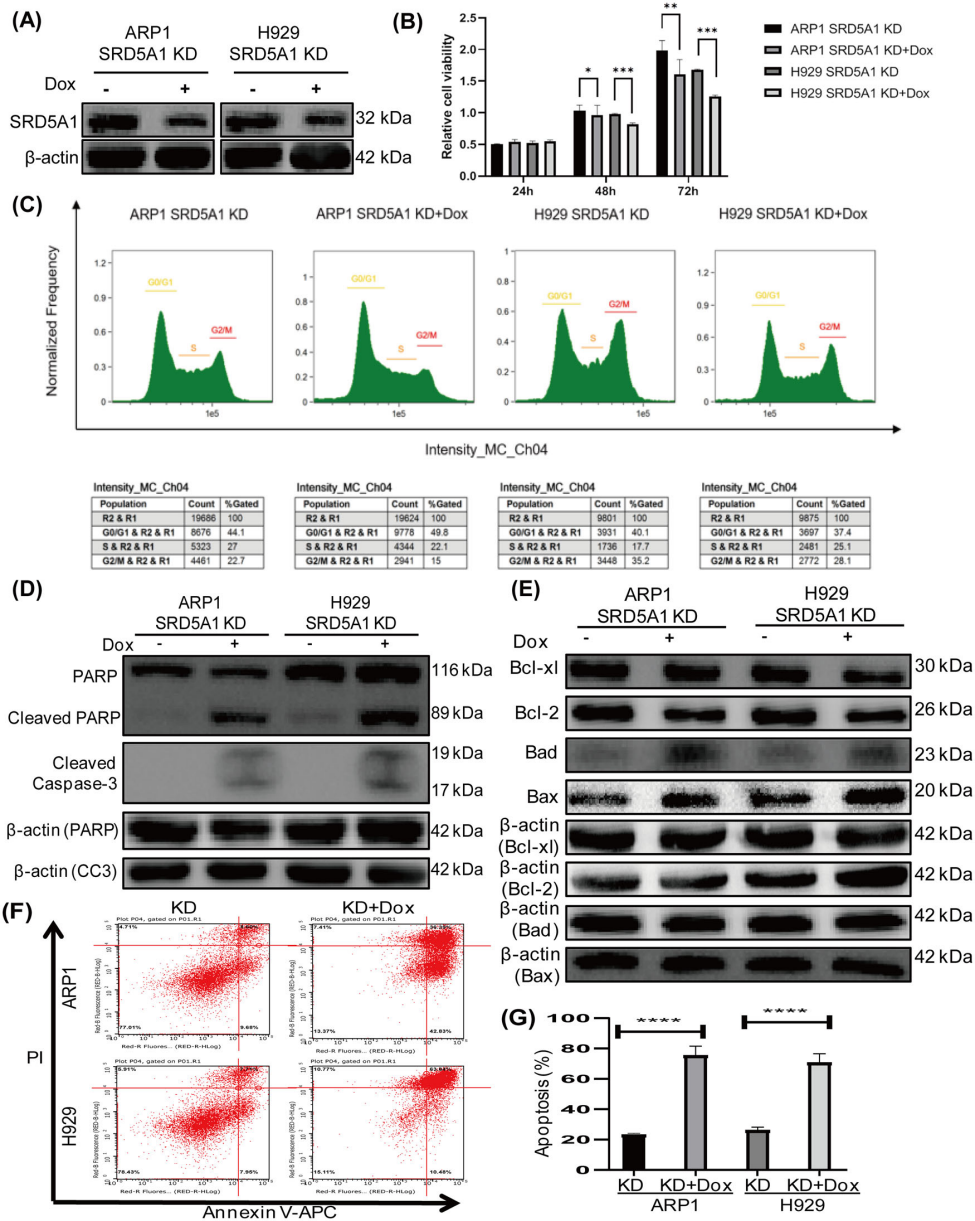
Inducible downregulation of SRD5A1 influences growth and survival of myeloma cells in vitro. **A** Western blotting analysis for SRD5A1 expression of ARP1- and H929-SRD5A1-KD cells treated with or without Dox. **B** Histogram plot depicted the growth of ARP1- and H929-SRD5A1-KD cells treated with or without Dox. **C** PI-staining cell cycle analysis of ARP1- and H929-SRD5A1-KD cells treated with or without Dox. **D** Western blotting revealed that SRD5A1 knockdown-induced apoptosis by detecting the expression of PARP and cleaved caspase-3 proteins. **E** Immunoblot analysis of Bcl-xl, Bcl-2, Bad, and Bax protein levels in ARP1- and H929-SRD5A1-KD cells treated with or without Dox. **F** Annexin-V/PI staining indicated that more apoptosis cells were induced post downregulation of SRD5A1 in ARP1 and H929 cells. **G** Quantification of apoptosis cells rate in (**F**).

To determine whether SRD5A1 functions as an oncogene in MM, we functionally knocked down SRD5A1 in myeloma cells by expressing the lentivirus-mediated SRD5A1-shRNA which was under the control of a doxycycline (Dox)-inducible gene promoter thus avoiding the adverse effect of long-term knockdown. Western blotting verified a remarkable downregulation of SRD5A1 expression in both ARP1 and H929 human MM cells after Dox treatment compared with nontreated cells (Fig. 2A). Both ARP1- and H929-SRD5A1-KD cells exhibited a significantly lower cell growth rate following 48 h of treatment with Dox (Fig. 2B).

To elucidate the diminished cell viability of SRD5A1-KD cells, cells were stained with PI to detect cell cycle by flow cytometry. As shown in Fig. 2C, the knockdown of SRD5A1 caused abnormal cell cycle arrest. We observed a similarly remarkable reduction of G2/M-phased cells of both ARP1 and H929, with a fluctuation of the G0/G1 and S phases in these two cell lines. In addition, the cell growth alteration may not be due to the cellular senescence, as few SA-β-gal-positive cells were found (Supplementary Fig. S2). Meanwhile, the decreased growth rate of SRD5A1-KD cells was ascribed to increased apoptotic cell death, which was evidenced by increased expression of cleavage of Poly ADP-ribose polymerase (PARP) and cleaved caspases-3 (Fig. 2D). We subsequently confirmed that knockdown of SRDA51 decreased the expression of Bcl-2 and Bcl-xl and increased the level of Bad and Bax (Fig. 2E). Consistently, the Annexin-V/PI staining assay by flow cytometry determined that more than threefold apoptotic cells were induced post by suppression of SRD5A1 (Fig. 2F, G). Therefore, transient knockdown of SRD5A1 could induce cell growth arrest and apoptosis in MM cells.

### Overexpression of SRD5A1 accelerates MM cell growth

**Fig. 3:**
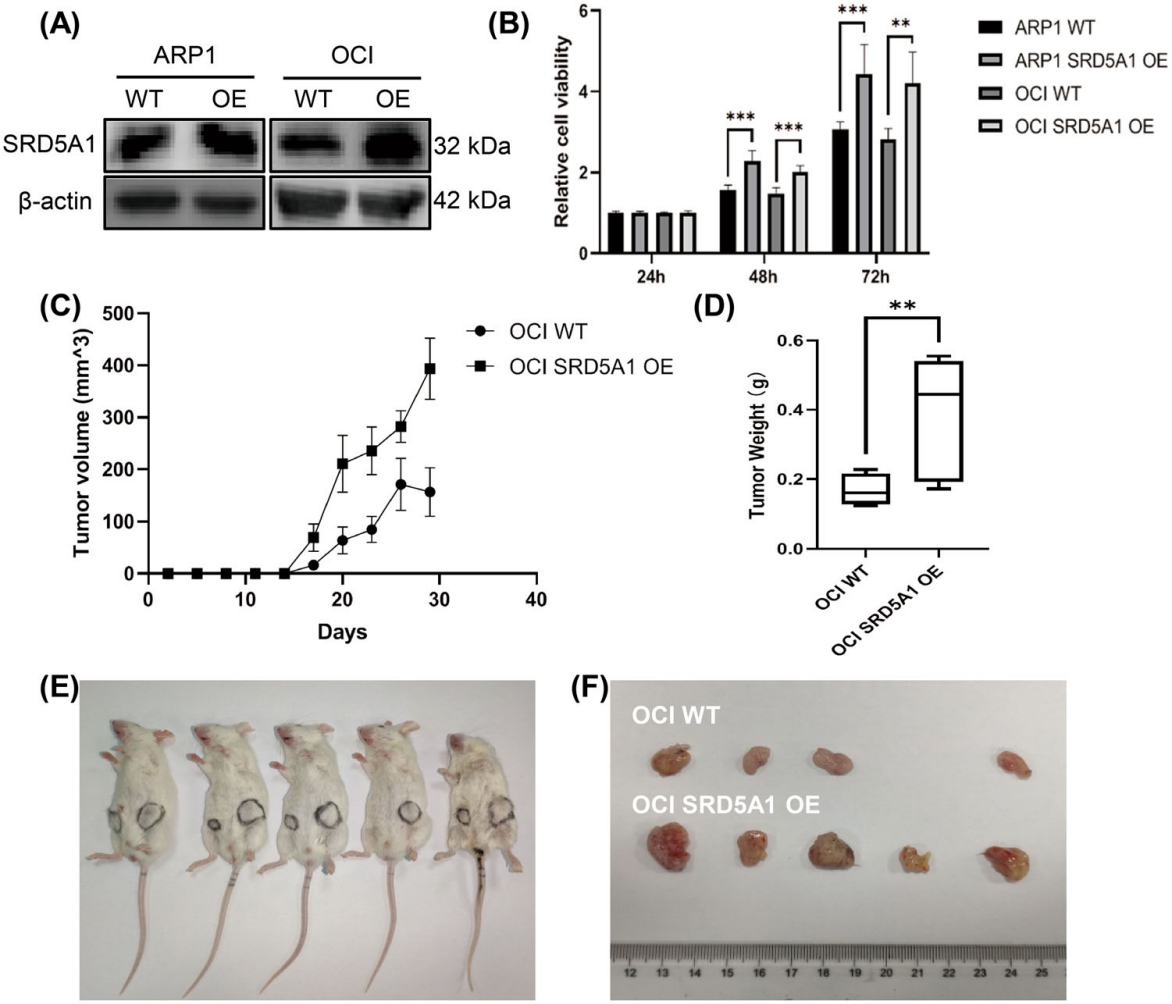
SRD5A1 overexpression promotes MM cellular proliferation in vitro and in vivo. **A** Western blotting analysis for SRD5A1 expression of WT and SRD5A1-OE cells. **B** Histogram plot depicted the growth of WT and OCI-MY5 SRD5A1-OE cells. **C** The oncogenic effect of SRD5A1 was examined in subcutaneous transplantation mouse models using OCI-MY5 WT and OCI-MY5 SRD5A1-OE cell lines. The result indicated that SRD5A1 significantly promoted OCI-MY5 tumor growth. **D** After treatment, the tumor weight of the SRD5A1-OE group was higher than that of the WT group. **E**, **F** The NOD-SCID mice injected subcutaneously with OCI-MY5 WT (left) and OCI-MY5 SRD5A1-OE (right) cells were sacrificed, and tumors were isolated. *P < 0.05, **P < 0.01, ***P < 0.001.

To further evaluate the impact of SRD5A1 on cell growth in vivo, we overexpressed SRD5A1 by lentivirus-mediated SRD5A1-cDNA transfection in the MM cell lines ARP1 and OCI-MY5. After assessing overexpression efficiency (Fig. 3A, B), we injected OCI-MY5 and OCI-MY5-SRD5A1-OE subcutaneously into the right and left flank of NSG mice. As shown in Fig. 3C–F, after about 30 days, the tumors derived from OCI-MY5-SRD5A1-OE cells were visibly bigger than their OCI-MY5 counterparts. The average weight of OCI-MY5-SRD5A1-OE tumors was lower than the control tumors. These results indicate that genetic overexpression of SRD5A1 facilitates myeloma in vivo, confirming the oncogenic roles of SRD5A1 in MM cells.

### Downregulation of SRD5A1 induces autophagy via regulating the PI3K/Akt/mTOR pathway in MM cells

**Fig. 4:**
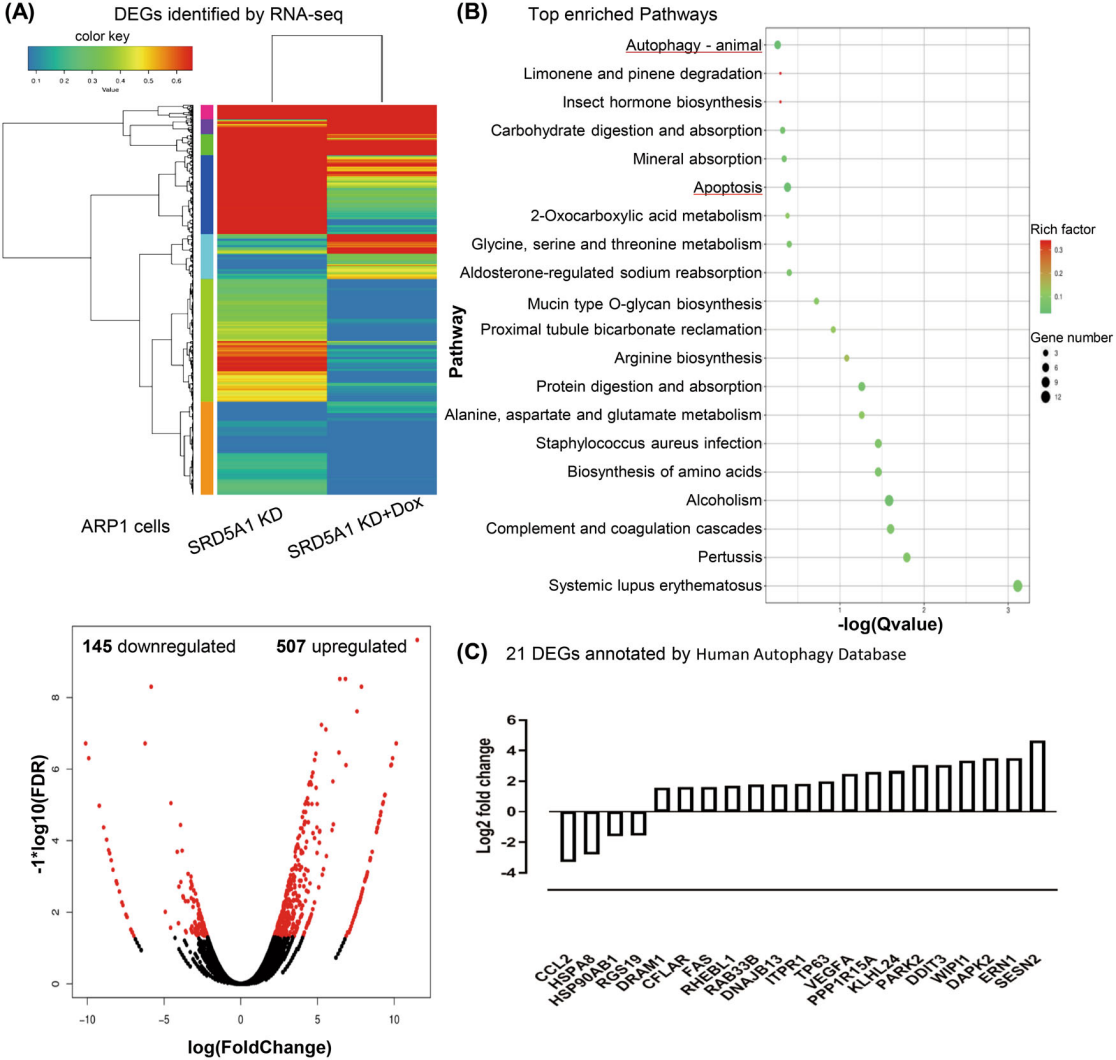
RNA sequencing results reveal differentially expressed genes (DEGs) and enriched KEGG pathways in MM cells after downregulating SRD5A1. **A** Transcriptiomic RNA-seq identified 652 DEGs. Hierarchical cluster analysis (top) of significantly differentially expressed mRNAs between ARP1-SRD5A1-KD cells treated with and without Dox. Volcano plot (bottom) showing 145 decreased genes and 507 upregulated genes. **B** Top enriched KEGG pathways for these 652 DEGs, including autophagy and apoptosis signaling. **C** Twenty-one DEGs (including four downregulated and 17 upregulated genes) could be annotated as the autophagy-related genes from Human Autophagy Database.

To further elucidate the mechanism, we prepared RNA samples for transcriptomic RNA sequencing (RNA-seq) to screen differentially expressed genes (DEGs) following the SRD5A1 knockdown. As shown in Fig. 4A–C, in a comparison between ARP1-SRD5A1-KD cells treated with or without Dox, the transcriptome analysis found 145 downregulated genes and 507 upregulated genes. The KEGG pathway enrichment of these DEGs hinted that SRD5A1 knockdown may significantly regulate many pathways, such as autophagy animals, apoptosis, mTOR signaling pathway, PI3K-Akt signaling pathway among the top 50 enriched pathways (with the top 20 enriched pathways shown in Fig. 4B). In addition, by querying Human Autophagy Database (http://www.autophagy.lu/index.html) with these above DEGs, we noticed that 21 (4 downregulated and 17 upregulated) could also be directly annotated as autophagy genes, indicating that SRD5A1 knockdown may be related to autophagic progress (Fig. 4C). Supporting this, we further examined gene expression profiling (GEP) of 22 normal people and 351 MM patients from the total therapy 2 (TT2) dataset, finding more autophagy genes with high expression than that of the low expression from the TT2 dataset (Supplementary Fig. S3A, B). Thus, we proposed that autophagy might be a target pathway influenced by SRD5A1 knockdown.

**Fig. 5:**
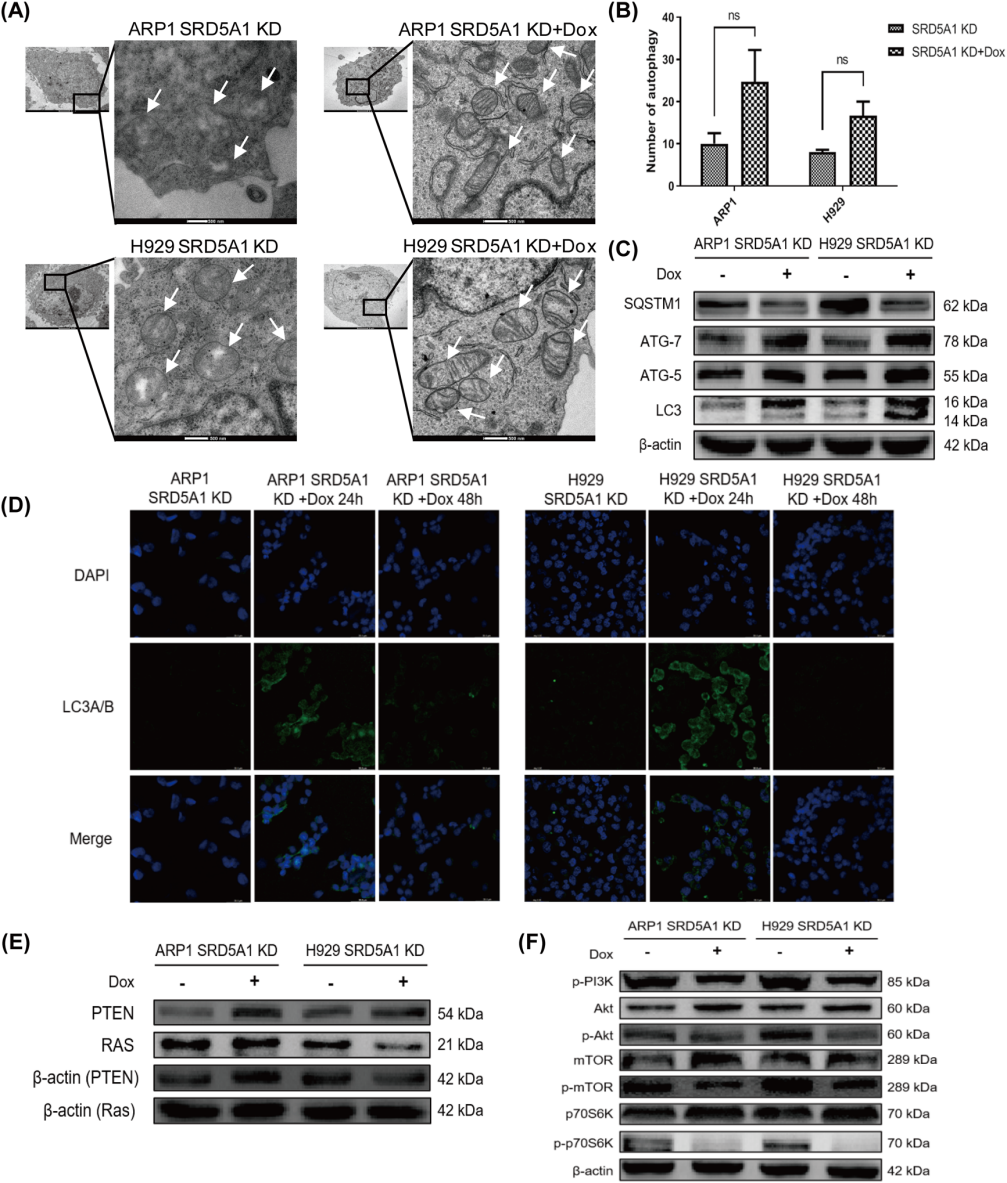
SRD5A1 knockdown induces autophagy via PI3K/Akt/mTOR pathway. **A** The ultrastructure of autophagosome in ARP1- and H929-SRD5A1-KD treated with Dox compared to SRD5A1-KD cells observed by transmission electron microscopy. **B** The level of autophagosome in (**A**) was quantified. One-way analysis of variance (ANOVA) was carried out. **C** Western blotting assay on SQSTM1, ATG-7, ATG-5, and LC3 expression in ARP1- and H929-SRD5A1-KD cells treated with or without Dox. **D** Immunofluorescence staining of LC3 expression in ARP1- and H929-SRD5A1-KD cells treated with or without Dox. **E** Western blotting assay on PTEN and Ras expression in ARP1- and H929-SRD5A1-KD cells treated with or without Dox. **F** Expression of a marker protein in PI3K/Akt/mTOR signaling was detected by western blotting in ARP1 and H929 cells.

To verify these findings, scanning electron microscopy was firstly utilized to evaluate autophagosomes and autophagolysosomes, finding that the number of them was only slightly increased in SRD5A1-KD myeloma cells compared to control (Fig. 5A, B). Next, western blotting confirmed the increased expression of several autophagy marker proteins including SQSTM1/p62, ATG-5, ATG-7 protein as well as LC3 conversion after SRD5A1 knockdown (Fig. 5C). In addition, the expression of LC3 proteins was also confirmed using immunofluorescence staining methods. As shown in Fig. 5D, LC3 reached peak expression after Dox treatment for 24 h in SRD5A1-KD MM cells. Therefore, we concluded that SRD5A1 knockdown-induced autophagy in MM cells.

The aforementioned KEGG pathway analysis suggested that SRD5A1 knockdown influenced the PI3K/Akt and mTOR signaling pathways. To test whether SRD5A1-KD induced autophagy via the PI3K/Akt/mTOR pathway, we first detected marker proteins in potential autophagy-related pathways, such as Ras and PTEN, but there was no apparent variation (Fig. 5E). However, another autophagy-related pathway was examined and shown in Fig. 5F that the phosphorylated protein levels of p-PI3K, p-Akt, p-mTOR, p-FoxO1, and p-p70S6K were obviously downregulated, whereas the protein level of Akt, mTOR, and p70S6K was elevated slightly or maintained unchanged in these cells following SRD5A1 knockdown. It indicated that knockdown of SRD5A1 may induce autophagy through regulating the PI3K/Akt/mTOR pathway.

### SRD5A1 knockdown-induced apoptosis could be aggravated by autophagy inhibitor 3-methyladenine in MM cells

**Fig. 6:**
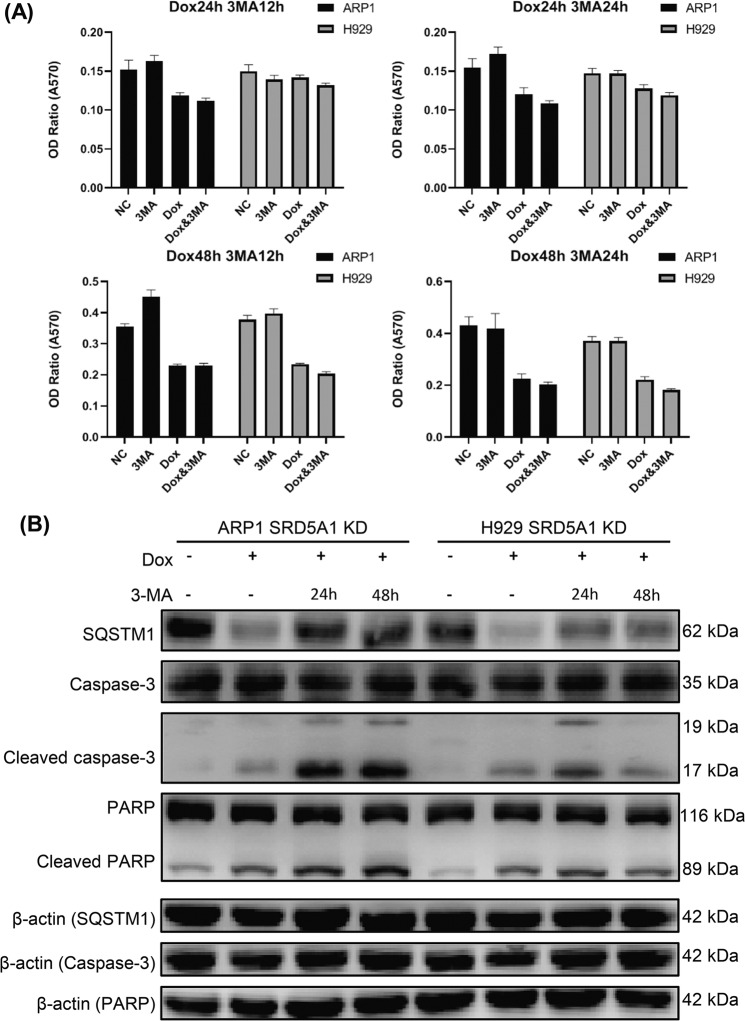
3-MA potentiates apoptosis induced by inhibition of autophagy. **A** Effect of 3-MA and/or Dox on the growth of ARP1 and H929-SRD5A1-KD cells measured by MTT assay. **B** Western blotting analysis of SQSTM1, caspase-3, cleaved caspase-3 and PARP, cleaved PARP in response to 3-MA and/or Dox treatment in ARP1- and H929-SRD5A1-KD cells. *P < 0.05, **P < 0.01, ***P < 0.001.

Autophagy and apoptosis are two essential self-destructive processes that regulate cell survival and death, and their relationship is complex because autophagy constitutes an adaptive response to diverse stress stimuli for avoiding cell death; yet, in some settings, it can also contribute to the demise of cells^11^. To test this, we first compared the impact of three different autophagy inhibitors, LY294002, hydroxychloroquine (HCQ), and 3-methyladenine (3-MA) on MM cell viability in a dosage range of 0.1–10^5^ nM. Results showed that 3-MA presented less inhibitory effect on cell viability compared to HCQ and LY294002 (Supplementary Fig. S4). Hence, we chose 3-MA to process SRD5A1-KD cells separately or with Dox jointly for different exposure times. Compared to the Dox group or 3-MA group for short treatment time (Dox 24 h and 3-MA 12 h), the MTT assay illustrated that the inhibitory effect of combined Dox and 3-MA was the most obvious at 24-h treatment with 3-MA meanwhile 48-h Dox induced SRD5A1 suppression (Fig. 6A).

Based on the results above, we proposed that 3-MA exerted its inhibitory effect on autophagy thus may potentiate apoptosis. The results of western blotting reinforced (Fig. 5B) expression of cleaved caspase-3 and cleaved PARP having a significant increase after treated with 3-MA for 24 h in SRD5A1-KD cells (Fig. 6B). Given all that, these results support the point that SRD5A1 knockdown-induced apoptosis could be aggravated by 3-MA mediated autophagy inhibition in MM cells, which could be applied as a novel therapeutic notion for cancer treatment.

### SRD5A1 inhibitor, Dutasteride, possesses a therapeutic effect on MM

**Fig. 7:**
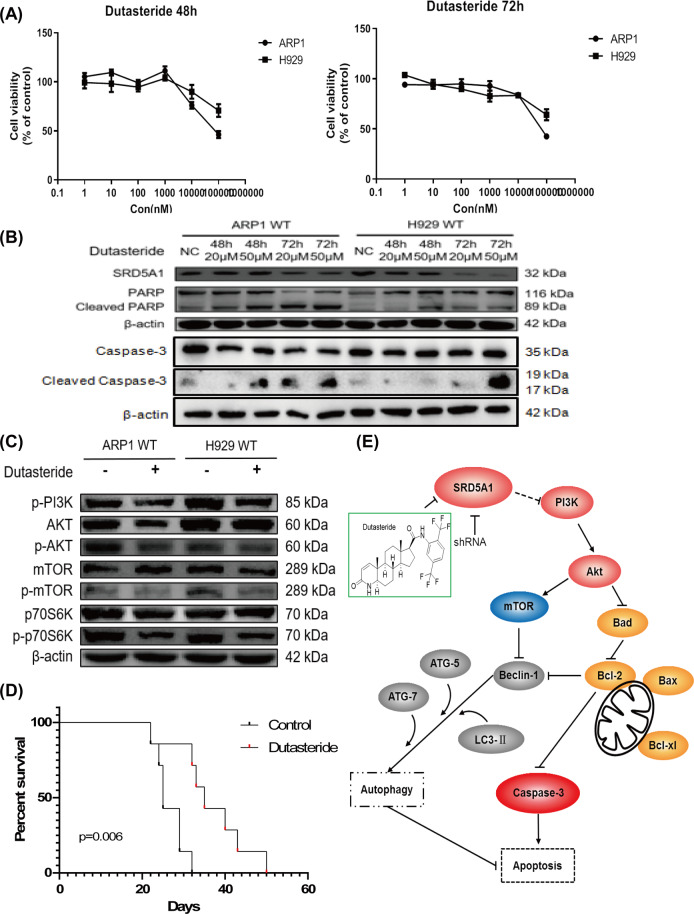
Dutasteride possesses a therapeutic effect on MM. **A** MTT proliferation assay of ARP1 and H929 cells treated with different concentrations of dutasteride for 48 or 72 h. **B** Expression of apoptosis markers in dutasteride-treated ARP1 and H929 cells as determined by western blotting analysis. **C** Western blotting showed that Dutasteride treatment inhibited the activity of PI3K/Akt/mTOR signaling in ARP1 and H929 cells. **D** An MM model was established by injecting 8-week-old C57BL/KaLwRij mice with intravenous 5TMM3VT via the tail vein (1 × 10^6^ cells per mouse; *n* = 7 per group). The survival curves of the 5TMM3VT mouse model with or without Dutasteride treatment are presented. **E** The model of our working hypothesis. Reduced SRD5A1 expression triggers apoptosis and autophagy activation eventually leads to cell death when autophagy cannot compensate for apoptosis-induced cellular stress.

To further explore the therapeutic utilization of the present preclinical study, we evaluated the effect of the SRD5A1 inhibitor Dutasteride on MM cell growth. As shown in Fig. 7A, Dutasteride dramatically reduced the viability of ARP1 and H929 cells, with IC50 at 78.446 μM and 17.156 μM, respectively. The western blotting results demonstrated that Dutasteride suppressed SRD5A1 expression in MM cells post treatment with two dosages (20 μM, 50 μM) for 48 h and 72 h. Meanwhile, we observed the increased expression levels of cleaved caspase-3 and cleaved PARP in a time- and dose-dependent manner (Fig. 7B). Consistent with the previous results in SRD5A1-KD cells (Fig. 5), the PI3K/Akt/mTOR signaling pathway was suppressed after Dutasteride treatment (Fig. 7C).

In order to further explore the antitumor effect of SRD5A1 inhibition, Dutasteride was intraperitoneally injected to MM mice (25 mg/kg body weight, three times/week) once the 5TMM3VT syngeneic MM-prone-model was established. Time-course analysis of tumor growth revealed that control MM mice rapidly deteriorated from day 22 until all mice were dead by day 32. However, Dutasteride-treated MM-prone mice exhibited prolonged survival rates up to 50 days (*P* = 0.0060; Fig. 7D). Therefore, downregulation of SRD5A1 outstandingly lagged the myeloma tumor growth in vivo.

## Discussion

Differing from necrosis, apoptosis is a highly programmed cell death process and is typically dysregulated in human cancers^12^. Massive apoptosis can induce the death of cancer cells, while aberrant apoptosis contributes to cancer initiation, progression, and treatment failure^13^. The specific molecular mechanism was uncovered in the 1990s, particularly specific genes regulating cell death in *Caenorhabditis elegans* and the B-cell lymphoma 2 (Bcl-2) was discovered as an arbitrator in apoptosis^14^. The Bcl-2 family controls apoptosis-mediated mitochondrial outer membrane permeabilization (MOMP) and includes the anti-apoptotic proteins (Bcl-2, Bcl-xL, and Mcl-1), pro-apoptotic effectors (Bak and Bax), and pro-apoptotic BH3-only proteins (Bid, Bim, Bik, Noxa, and Puma)^15,16^. The executor of apoptosis caspase activation results in MOMP, thereby releases apoptotic components, such as cytochrome c, Smac, and Omi. Nowadays targeting apoptosis has been increasingly recognized as a promising approach to kill cancer cells^17^. Our results detecting changed Bcl-2 family proteins in Figs. 2 and 7 strongly support that inhibition of SRD5A1 by either RNAi or drugs (Dutasteride) treatment facilitates apoptosis instead of necrosis or cellular senescence in MM cells. Although Bcl-2 family proteins are initially regarded as regulators of cell death, they also participate in autophagy by constitutively interacting with Beclin-1, which promotes autophagy and inhibits tumorigenesis in mammalian cell^18,19^.

The term “autophagy” was coined by Christian de Duve at the Ciba Foundation Symposium on Lysosomes for the first time in groundbreaking work on the discovery of lysosomes in the 1950s, but massive researches began in 2008 (refs. ^20,21^). Recent reports indicate that autophagy plays a dichotomous role in tumorigenesis: extravagant autophagy triggers cancer cells to “autophagic cell death” (ACD) through excessive self-digestion and degradation of essential cellular constituents^22^, while more often, under normal physiological conditions, autophagy functions as a patron to counteract with cancer by eliminating damaged organelles and recycling degradation products^23^. Autophagy is observed at a higher frequency in cancer cells than in normal cells, and some cancers even count on autophagy for survival under external stresses such as starvation, hypoxia, growth factor withdrawal, chemotherapy, or radiotherapy^24,25^. In this study, SRD5A1 knockdown-induced autophagy existed merely in the beginning phase after treatment of Dox (before 24 h) and vanished at 48 h according to our observation (Figs. 5D and 6A). Hence, we suspected autophagy might be an attempt of MM cells for survival, so we went on to verify that inhibiting autophagy by 3-MA could contribute to cell death (Fig. 6B).

However, the cross-talk between apoptosis and autophagy is complex and still vague, as synergistic, antagonistic, and interdependent effects have been observed^26^. Normally, autophagy precedes apoptosis and maintains homeostasis, as reflected by that inhibition of autophagy leads to accumulation of damaged and harmful cellular constituents, which in general increases cellular stress levels and activates sensitivity against programmed cell death^22^. Programmed cell death, such as apoptosis, is triggered once cellular stress is prolonged for a critical duration or exceeds the intensity threshold. Several studies have reported that these two highly conserved processes are tightly regulated by overlapping components and can interact with each other in many types of cancers, such as Beclin-1/Bcl-2 and FLIP (FADD-like IL-1b-converting enzyme-inhibitory protein), autophagy-related proteins (ATGs), caspases^27^. Targeting apoptosis has been the overwhelming focus of methods aimed at killing cancer cells, and experimental evidence shows that autophagy participates in resistance to chemotherapy-induced cell death^28^. In MM cells, the abnormal autophagy could regulate proliferation and apoptosis, which advances the progress of MM^29,30^. In this study, SRD5A1 suppression simultaneously induced apoptosis through Bcl-2 family proteins as well as autophagy via PI3K/Akt/mTOR signaling, meanwhile, the autophagy inhibition using 3-MA potentiated MM cell apoptosis. Consistent with our findings, one recent study report that silencing of the stress-related nuclear protein 1 (NUPR1) could suppress autophagy activities and induces autophagy-mediated apoptosis in two MM cell lines (U266 and RPMI 8226) through the PI3K/AKT/mTOR pathway^31^. In fact, the autophagy inhibitor drug chloroquine could also potentiate carfilzomib-induced apoptosis in myeloma cells in vitro and in vivo^32^. Therefore, the above findings support the therapeutic utilization of trials targeting both autophagy and autophagy-mediated apoptosis to improve anti-MM therapy^33^.

To the best of our knowledge, this study for the first time established a link between the dual-autophagy–apoptosis-regulatory SRD5A1 and the activity of MM. SRD5A1, which maps to a previously defined amplicon at 5p15.31, catalyzes the conversion of testosterone into the more potent androgen, dihydrotestosterone (DHT), which was significantly overexpressed in newly diagnosed MM patients with a short survival^34,35^. Elevated levels of SRD5A1 have been reported to be correlated to the severity of multiple cancers with their nidus situated to the parts rich in sex hormone, including prostate cancer, breast cancer, endometrial cancer, or the like, and to facilitate cell proliferation^32^. Nonetheless, neither testosterone nor DHT has an impact on myeloma cell proliferation in this study (data not shown), but we did find that varying levels of SRD5A1 could alter the growth of myeloma cells. It may be mainly due to the oncogenic role of SRD5A1 in these tumor cells including MM and other prostate cancers (Fig. 1). However, we cannot exclude the indirect roles of its enzymatic substrate–androgens in the MM pathogenesis. Although a recent study indicated that SRD5A1^−/−^ mice had reduced bone mass and diminished areal bone mineral density without changed circulating levels of androgens in serum, it may be explained by the compensatory adjustment of steroids metabolism among SRD5A1, SRD5A2, SRD5A3 (ref. ^36^). In fact, it is evidenced that tumor development is relevant to the metabolism of steroids within the local tumor and its adjacent host tissue which might create a microenvironment to promote the cancer^37^. Besides, the effects of testosterone can also be exerted through aromatization to estrogens, which could exert their carcinogenic effects through binding with estrogen receptors^38^. There are two 5α-reductase inhibitors tested clinically with Finasteride specifically inhibiting SRD5A2 and Dutasteride targeting both SRD5A1 and SRD5A2^39,40^. We also presented the therapeutic effect of SRD5A1 inhibitor Dutasteride on MM (Fig. 6). Further preclinical studies are needed to evaluate the detailed mechanism as well as its therapeutic potential in vivo. Therefore, the results presented herein characterize a novel mechanism by which SRD5A1 can be a novel target in MM, and it is urgent to develop more novel agents specifically targeting SRD5A1.

Taken together, we discovered the previously unclear role of SRD5A1 in MM. SRD5A1 simultaneously regulates both apoptosis mediated by Bcl-2 proteins family and autophagic process via PI3K/Akt/mTOR signaling (Fig. 7E). On the basis of this dual autophagy–apoptosis regulatory potential of SRD5A1, we propose that SRD5A1 may be a potential novel target for MM.

## Supplementary information

Supplementary information

## Data Availability

All datasets generated during this study are included in this published article including its Supplementary Files and original RNA-seq datasets deposited in NCBI Gene Expression Omnibus (GSE155858).
