# Supplementary material for: Suppression of steroid 5α-reductase type I promotes cellular apoptosis and autophagy via PI3K/Akt/mTOR pathway in multiple myeloma
Source: Cell Death Dis. 2021 Feb 24;12(2):206. doi: 10.1038/s41419-021-03510-4 (PMC7904855; doi:10.1038/s41419-021-03510-4)
Supplement: Supplementary file 1 — Supplementary information [file 41419_2021_3510_MOESM1_ESM.doc]

**Supplementary information**

**Supplementary Figure 1-4**

**
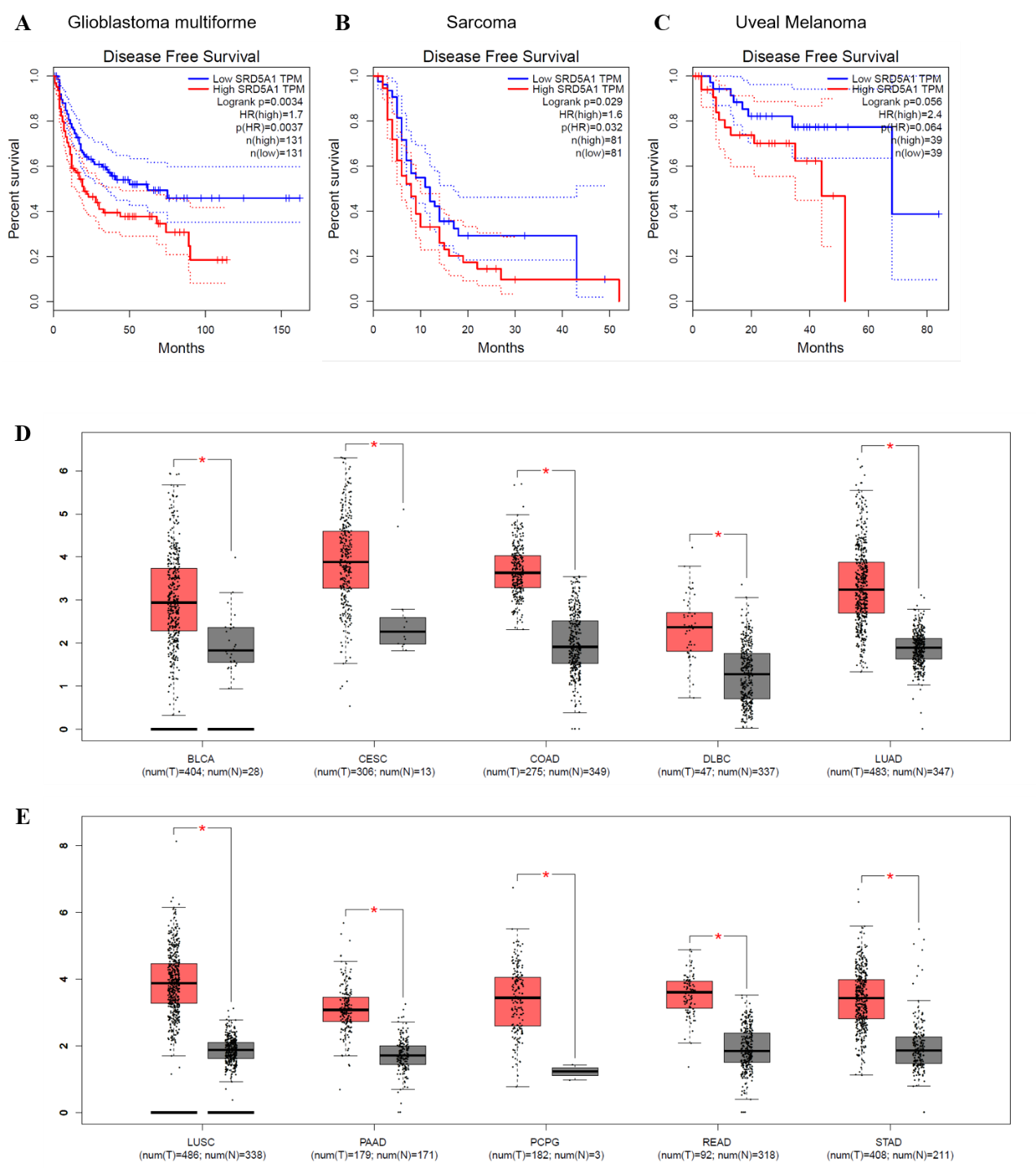
**

**Figure S1.** **SRD5A1 correlates to prognosis in multiple cancers.** (A&B) Disease Free Survival of glioblastoma multiforme, sarcoma and uveal melanoma. (C-E) Boxplot of SRD5A1 expression in bladder urothelial carcinoma, cervical squamous cell carcinoma and endocervical adenocarcinoma, colon adenocarcinoma, lymphoid neoplasm diffuse large B-cell lymphoma, lung adenocarcinoma, lung squamous cell carcinoma, pancreatic adenocarcinoma, pheochromocytoma and paraganglioma, rectum adenocarcinoma, stomach adenocarcinoma and their counterparts.


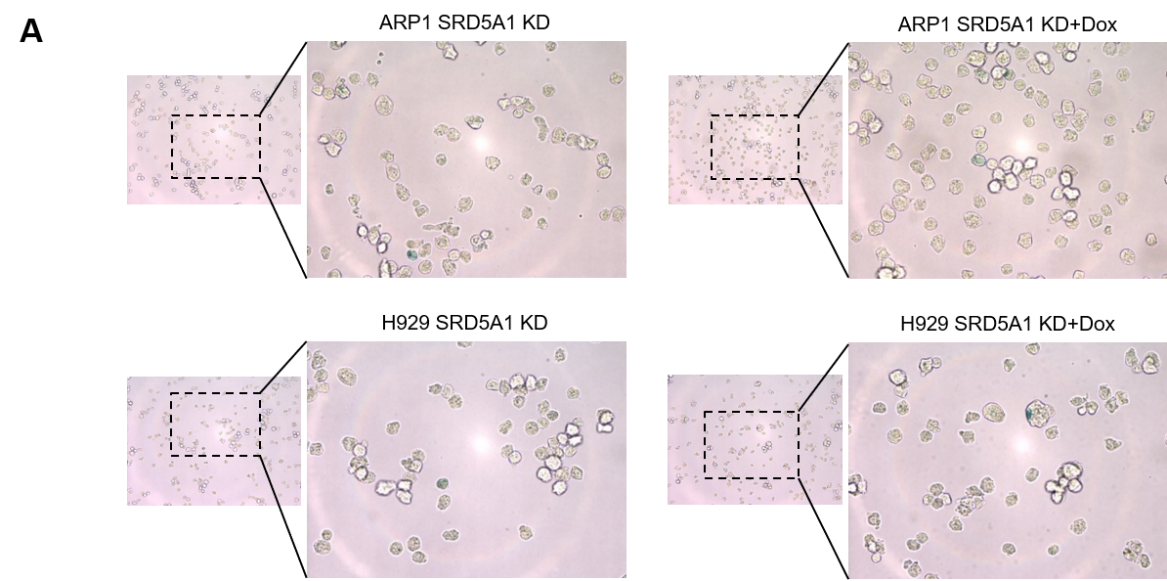


**Figure S2.** **The cell growth arrest is not related to cellular senescence** **in SRD5A1-KD cells.** SA-b-galactosidase (Green) cellular senescence staining of ARP1- and H929-SRD5A1-KD treated with Dox (right panel) compared to SRD5A1-KD cells (left panel).


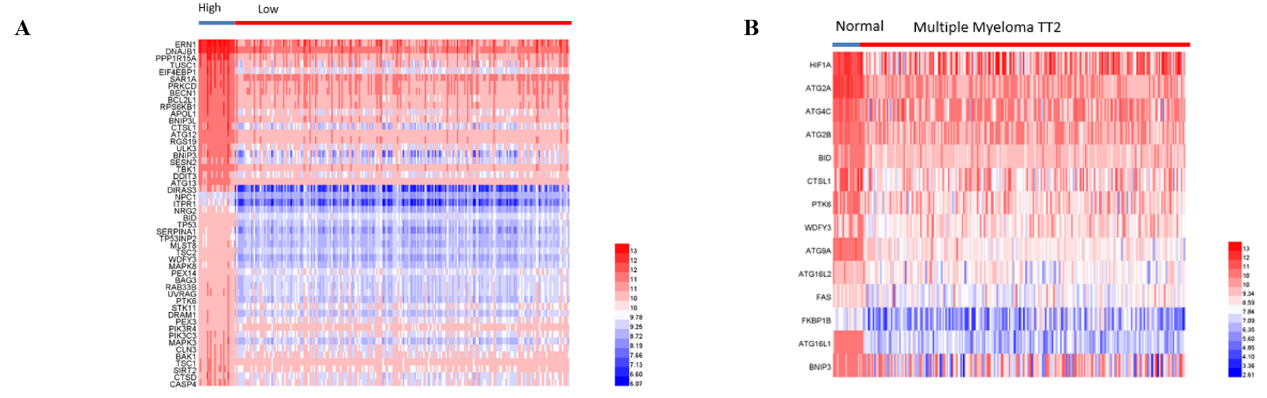


**Figure S3. A number of autophagy genes are highly expressed in TT2 dataset.** (A) Heatmap of the 50 differentially expressed genes correlated with autophagy. (B) Gene expression clustergram of 14 autophagy genes in plasma cells from 22 normal people and 351 MM patients.


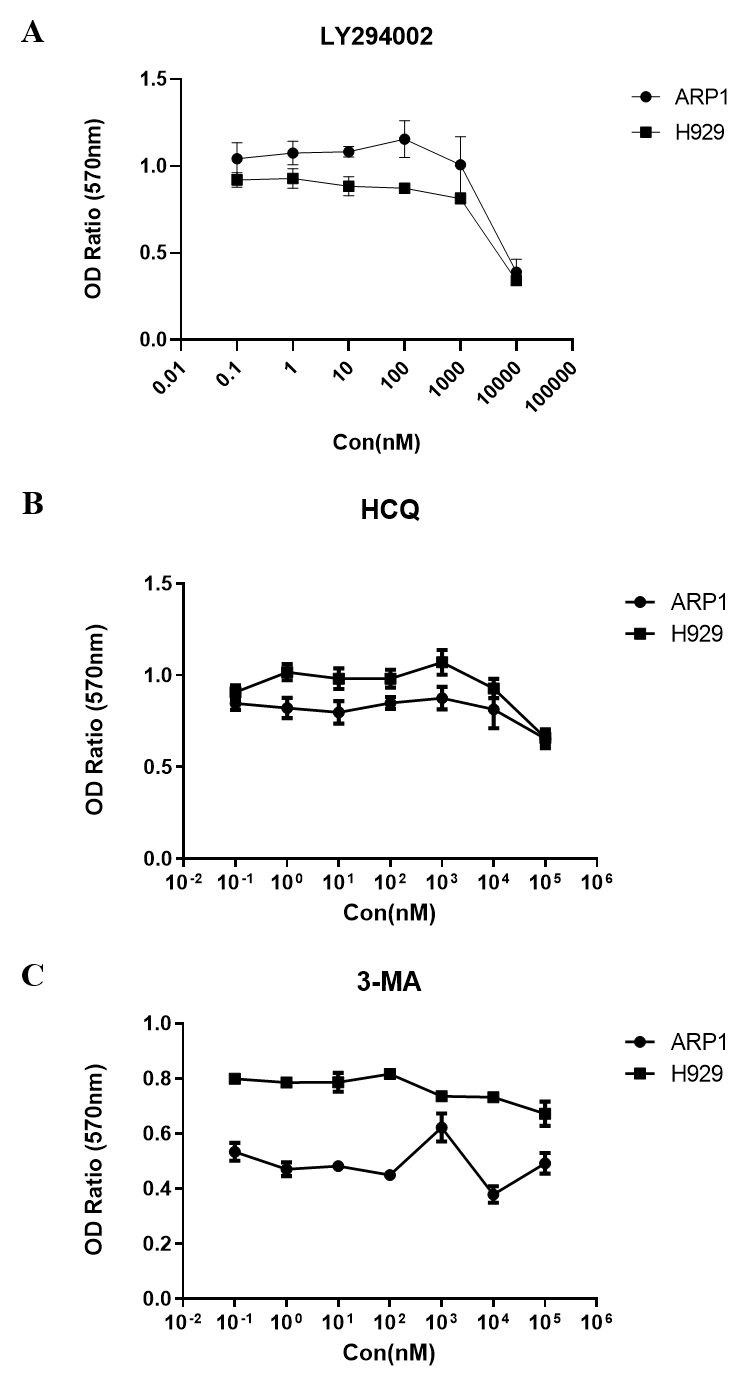


**Figure S4.** **MTT assays validate the effect of different autophagy inhibitors on the growth of ARP1 and H929 cells.** (A) LY294002; (B) HCQ; (C) 3-MA.
